# The Risk of COVID-19 Pandemic in Patients with Moderate to Severe Plaque Psoriasis Receiving Systemic Treatments

**DOI:** 10.3390/vaccines8040728

**Published:** 2020-12-02

**Authors:** Paolo Gisondi, Francesco Bellinato, Andrea Chiricozzi, Giampiero Girolomoni

**Affiliations:** 1 Section of Dermatology and Venereology, Department of Medicine, University of Verona, 37126 Verona, Italy; paolo.gisondi@univr.it (P.G.); giampiero.girolomoni@univr.it (G.G.); 2 Institute of Dermatology, Catholic University, 00185 Rome, Italy; andrea.chiricozzi@unicatt.it

**Keywords:** psoriasis, Sars-CoV-2, COVID-19, biologics, synthetic and biological disease-modifying antirheumatic drugs (DMARDs)

## Abstract

Chronic plaque psoriasis is an inflammatory skin disease affecting 2–3% of the general population. Approximately one-third of patients are candidates for systemic immunosuppressive treatments, such as synthetic or biological disease-modifying antirheumatic drugs, because of disease extensions, localization in sensitive or visible areas and/or resistance to topical treatments. These therapies have been associated with increased risk of infection, including upper respiratory tract viral infection. Psoriasis is frequently associated with cardio-metabolic comorbidities, such as obesity and diabetes, that are risk factors for poor prognosis in the case of coronavirus disease (COVID-19) pneumonia. A narrative review of the literature based on an electronic search of the PubMed^®^ database was undertaken with the objective of investigating whether there is an increased risk of COVID-19 infection in psoriasis patients on systemic treatment. Original articles, such as case reports, published up to 1 November 2020 were included. There is no evidence that patients with moderate-to-severe psoriasis receiving systemic treatments, including biologics, have higher risk of SARS-CoV-2 infection and/or increased hospitalization and death related to COVID-19 compared to the general population. Several case reports described full recovery from COVID-19 with favorable outcomes in psoriasis patients who were being treated with synthetics or biologicals. Nonetheless, caution should be maintained in this setting, and more data are needed to draw definitive conclusions.

## 1. Introduction

**Figure 1:**
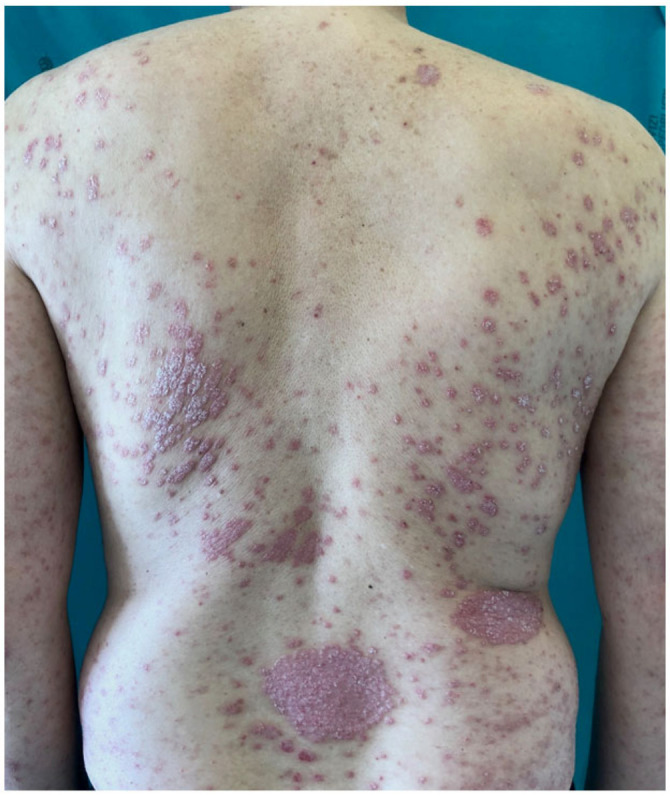
Man affected by severe psoriasis, a candidate for systemic treatment.

The pandemic of coronavirus disease (COVID-19), a viral pneumonia caused by severe acute respiratory syndrome coronavirus 2 (SARS-CoV-2), has raised serious concern among dermatologists and psoriatic patients receiving systemic immunomodulating/immunosuppressive treatments [1]. Plaque psoriasis is a chronic inflammatory skin disease affecting 2–3% of the general population, and approximately one-third of patients are candidates for systemic treatments because of disease severity, extensions and/or localization in sensitive or visible areas (Figure 1) [2]. Systemic treatments include synthetic or biologic disease-modifying antirheumatic drugs (DMARDs), such as methotrexate, cyclosporine and TNF-α, IL-17, IL-12/23 and IL-23 inhibitors, which have been associated with increased risk of infection, including respiratory tract viral infection [3]. Moreover, patients with moderate-to-severe psoriasis are also frequently affected by cardio-metabolic comorbidities, such as obesity, diabetes mellitus and arterial hypertension, that have been associated with higher risk of hospitalization and fatal outcome of COVID-19 pneumonia [4]. The aim of this narrative review was to investigate whether there is an increased risk of COVID-19 infection or more severe infection outcomes in psoriasis patients on systemic treatments.

## 2. Materials and Methods

A narrative literature review based on an electronic search of PubMed^®^ was performed. The key words used were (“Sars-CoV2 infection” OR “COVID-19”) AND (“psoriasis”, “plaque psoriasis”, “biologic”, “methotrexate”, “cyclosporine”, “acitretin”, “dimethyl fumarate”, “apremilast”, “TNF-α inhibitors”, “IL-17 inhibitors”, “IL-12/23 inhibitors” OR “IL-23 inhibitors”). Two investigators (PG, FB) independently extracted data, and two other authors (AC, GG) were consulted to resolve any disagreement. A total of 57,243 articles were screened by title and abstract, and those deemed relevant were reviewed in full text and selected or rejected based on the inclusion and exclusion criteria. Original articles and reviews published up to 1 November 2020 assessing the risk of Sars-CoV-2 and/or COVID-19 infection and reporting the clinical outcome in patients with chronic plaque psoriasis and/or psoriatic arthritis receiving systemic treatment were retrieved. A total of 27 references were analyzed and 57,216 were excluded. For each manuscript, the following information was collected: type of study; sample size; data extraction method (e.g., registry-based, telephone call-based); type of systemic treatment, including synthetic and biological DMARDs; presence of inflammatory diseases other than psoriasis that require systemic treatment (e.g., Crohn’s disease, uveitis and hidradenitis suppurativa); clinical course of COVID-19. Suspected, but not confirmed, cases of COVID-19 were excluded. As measures of the severity of COVID-19 clinical outcomes, the following issues were considered: hospitalization, need of intensive care unit (ICU)-level care and death.

## 3. Results

### 3.1. Susceptibility to COVID-19 Infection in Psoriasis Patients Taking Immunomodulatory/Immunosuppressive Drugs

Whether psoriasis itself could confer susceptibility to SARS-CoV-2 virus infection is not known. At this stage, no studies have investigated the prevalence or incidence of SARS-CoV-2 infection in asymptomatic patients with plaque psoriasis. Those with psoriasis are likely exposed to the SARS-CoV-2 virus according to the spreading of the pandemic where they live. By contrast, different studies have assessed the incidence of SARS-CoV-2 infection in patients with plaque psoriasis receiving systemic treatments (Table 1). A Spanish study based on a national, multicenter, prospective cohort registry estimated no significant difference in standardized incident ratio (SIR) for SARS-CoV-2 infection in psoriatic patients treated with systemic therapies compared to the general population (SIR = 1.58; 95% CI 0.98–2.41) [5]. In a cohort study of 1830 patients from the Veneto Region (Northeast Italy), we found similar incidence of COVID-19 infection in psoriasis patients on biologics compared to the general population (IR = 9.7; 95% CI 3.9–20.1 per 10,000 person-months versus 11.5; 95% CI 11.4–11.7 per 10,000 person-months, respectively) [4]. A cohort study from two provinces in Northern Italy involving 246 psoriatic patients receiving biologics found that only one patient was tested and was positive for SARS-CoV-2, developing no symptoms during the observation period [6]. Only in a smaller study of 139 patients in Bergamo area was a higher prevalence of Sars-CoV-2 infection reported compared with the general population (3.6% versus 0.7%) [7]. However, the significance of this study is questionable, given the small number of cases and the fact that 2 out of 5 reported cases were not tested for COVID-19.

Large cohort studies have assessed the risk of hospitalization, intensive care unit admission and mortality due to COVID-19 in psoriasis patients treated with systemic therapies, mostly biologics (Table 2). We initially investigated the risk of hospitalization and death from COVID-19 in patients with chronic plaque psoriasis receiving biologics and renal transplant recipients in maintenance immunosuppressive treatment. No hospitalization or death was documented in 980 patients with psoriasis on biologics, and only one kidney-transplanted patient was hospitalized among 280 patients in follow up between February and April 2020 [8]. Then, a retrospective multicenter observational study including 5206 patients from six provinces of North Italy treated with biologics was reviewed. Similarly, no cases of death from COVID-19 were found between February and April 2020 (IR = 0; 95% CI 0–5.1 compared with 1.6 per 10,000 person-months in the general population), and only four patients were hospitalized for COVID-19 interstitial pneumonia (IR = 5.6; 95% CI 1.5–14.3 compared with 5.9 per 10,000 person-months in the general population) [9]. Another cohort study from the Veneto region confirmed this finding. IRs of hospitalization and death for COVID-19-related pneumonia of 6.5 (95% CI 2.0–15.6) and 0 (95% CI 1–10.4) per 10,000 person-months, respectively, were found, compared to 9.6 (95% CI 9.4–9.7) and 1.16 (95% CI 1.10–1.21) per 10,000 person-months in the general population [4]. A single-center case–control study from Lombardy reported only five hospitalizations and no deaths out of 1193 psoriatic patients treated with biologics or small molecules. Although patients on biologics were at higher risk of hospitalization with respect to the general population of Lombardy, with an OR of 3.59 (95% CI 1.49–8.63), no increased risk of intensive care unit admission or death was found. This study indicates that patients on biologics are at higher risk of SARS-CoV-2 infection even though the severity of COVID-19 may not increase [10]. Similar studies from other countries confirmed these results. An American retrospective cross-sectional study including 412 patients receiving systemic immunomodulatory medications for cutaneous diseases, including psoriasis, found an infection rate and COVID-19 outcomes similar to those found in the general population, reporting only five infections and one hospitalization. The authors concluded that the risk of COVID-19 infection and the risk of poor outcomes are minimally affected by dermatologic immunomodulatory medications [11]. Consistent findings were reported also by a French multicenter, cross-sectional study involving 1418 patients receiving systemic treatment, including methotrexate, cyclosporine, acitretin, apremilast and biologics. A total of five (0.35%) patients had a severe form of COVID-19 requiring hospitalization, although 60% of them presented with other risk factors for severe infection [12].

Updated and open-access data about the impact of COVID-19 pneumonia on psoriasis patients are currently available in the PsoPROTECT (Psoriasis Patient Registry for Outcomes, Therapy and Epidemiology of Covid-19 infecTion) registry. PsoPROTECT is a global initiative that aims to identify predictors of COVID-19 outcomes in psoriasis and to characterize patients in whom it may be beneficial to pause, continue or initiate systemic treatment. Registry data from PsoPROTECT appear reassuring and do not show either an increased risk of SARS-CoV-2 infection or severe COVID-19 courses in psoriasis patients, including those on systemic therapies [13].

### 3.2. Course of COVID-19 Infection in Psoriasis Patients Receiving Systemic Treatments 

Studies reporting the course of COVID-19 in psoriasis patients receiving systemic treatments are summarized in Table 3. Mahil et al. analyzed the course of COVID-19 in 374 clinician-reported psoriasis patients from 25 countries, including patients on biologics (71%), non-biologics (18%) and no systemic treatment (10%) [14]. A total of 348 patients (93%) fully recovered from COVID-19, 77 (21%) were hospitalized and 9 (2%) died. The authors found that biologic systemic therapies were associated with lower risk of COVID-19-related hospitalization, compared with non-biologic systemic therapies [14]. Finally, a retrospective cohort study on 104 patients affected by psoriasis and polymerase chain reaction (PCR)-confirmed COVID-19, including patients treated with synthetic (cyclosporin and methotrexate) and biological DMARDs, found no significant differences in COVID-19 outcomes between patients taking or not taking systemic therapies [15]. Data retrieved from case–control studies, cohort studies and case reports showed full recovery form COVID-19 after synthetic and biologic DMARDs treatment interruption. Rarely these patients required hospitalization [16,17,18,19,20,21]. Favorable outcomes of COVID-19 infection in patients who continued apremilast and certain biologics, such as adalimumab, ustekinumab, ixekizumab and guselkumab, were also reported [6,22,23,24,25,26].

## 4. Discussion

The major finding of this review is that, apparently, there is no increased susceptibility for SARS-CoV-2 infection or increased severity of the disease course of COVID 19 in patients with psoriasis receiving synthetic or biologic DMARDs. This finding may appear surprising because systemic treatments have been associated with higher risk of other infections, as cytokines inhibited by biologics are involved in the immune response against pathogens. The influence of biologics on respiratory tract infection susceptibility has recently been investigated. Higher risks of opportunistic infections and herpes zoster were reported among patients receiving immunosuppressive therapy to treat moderate-to-severe psoriasis [27]. TNF-α is expressed in the lung epithelial cells in response to respiratory viruses, such as influenza, and it recruits lympho-monocytes in the site of infection. Nevertheless, no higher serum levels of TNF-α in severe acute respiratory syndrome (SARS) were found, compared to other respiratory illness [28]. A meta-estimate from phase 3 placebo-controlled clinical trials of anti-TNF-α in psoriasis found no evidence of an increased risk of respiratory tract infection (RTI) (OR 1.08; 95% CI 0.84–1.38) [29]. A similar meta-estimate on anti-IL-17 agents found an increased risk of RTI of any etiology (OR 1.56; CI 95% 1.04–2.33) [30]. A pooled analysis of clinical trials of secukinumab versus etanercept found a similar rate of RTI [31]. IL-17A in viral infections may contribute to secondary inflammatory injury recruiting neutrophils and lymphocytes, and its levels have been found to be elevated in patients with severe COVID-19 pneumonia [32]. A slightly reduced risk of respiratory infections among patients treated with ustekinumab versus secukinumab was reported in a Swedish population-based register-linked cohort study [33]. Data from pharmacovigilance indicate that anti-IL-23 biologics are not associated with additional risk of infection. Similar rates of infection were found in patients treated with guselkumab and risankizumab, respectively, compared to adalimumab [34,35]. The risk of infection due to conventional immunosuppressant drugs has been extensively investigated. Cyclosporin is well known to be associated with an increased risk of infection [36]; a multicenter prospective cohort study found a 58% higher risk compared with methotrexate (adjusted RR 1.58; 95% CI 1.17–2.15) [37]. Nevertheless, cyclosporin targets cyclophilin, which is required for viral replication, and it may inhibit influenza A virus, hepatitis C virus and coronavirus [38]. Methotrexate is also associated with increased risk of infection; a multicenter prospective cohort study reported a 40% higher risk of infection compared to acitretin [37]. Acitretin does not appear to cause immunosuppressive adverse events; a study from the BIODAVERM registry comparing the infection rates among different systemic drugs found that acitretin showed the lowest risk of infection [37]. 

Apremilast does not affect B cells, T cells, or IgG and IgM secretion, but it partially inhibits TNFα, INF-γ, IL-17 and IL-23. Given its immunomodulatory properties and its specific mechanism of action, refs. [14,16] apremilast does not favor either infection or cytokine storm, and it does not increase the risk of pulmonary fibrosis, one of COVID-19’s mortality factors. 

In addition to the potential treatment-associated risk of infection, psoriatic patients show higher prevalence of cardio-metabolic comorbidities compared to the general population. Severe COVID-19 patients are older and affected by comorbidities such hypertension and diabetes mellitus [39]. Hypertension, diabetes mellitus and cardiovascular disease are more prevalent in patients with psoriasis and are associated with risk of hospitalization and fatal outcome of COVID-19 pneumonia [4]. The potential impact of co-morbidity status such as obesity, hypertension, diabetes and positive history of cardiovascular disease was addressed in the international study of Mahil et al. analyzing data from the PsoPROTECT registry. They found that risk factors including being older, male, non-white and having chronic lung disease were associated with higher hospitalization rates for COVID-19 [14]. Some hypotheses may explain why a higher incidence of fatal COVID-19 outcomes was not found in psoriasis patients on systemic treatments. Patients with psoriasis under systemic treatment may have more defensive behaviors (e.g., wearing a facial mask, social distancing) compared to the general population. Another hypothesis relies on a possible protective effect against COVID-19 provided by biologics. Inhibition of the COVID-19 immune response would be harmful in the early phase of infection, but it would be helpful in the progression to the severe form of the disease [30]. In COVID-19, inflammatory cytokines appear to play a double role: they may elicit the activation of the immune response in the first phase, but later they can mediate the development of an exaggerated systemic inflammation [30]. Because of the hyperactive inflammatory effects of SARS-CoV-2, agents that modulate the immune response are being explored as adjunctive treatments for the management of moderate to critical cases. There is sufficient evidence to support clinical trials of anti TNF-α therapy in patients with COVID-19 [40,41]. It was recently demonstrated that angiotensin-converting enzyme 2 (ACE2) is the main receptor employed by SARS-CoV-2. Because IL-17 mediates inflammation, increasing ACE2 expression in epithelia, IL-17 inhibitors may prove advantageous in patients with psoriasis at risk of infection [42]. In addition, given that COVID-19 pneumonia patients with a Th17 profile may show worse outcomes, a clinical trial investigating the use of anti-IL17 is currently ongoing [43,44]. Nonetheless, there is concern that patients on immunosuppressant biologic therapies might be at higher risk of being infected and a question as to whether they need to discontinue their treatment pre-emptively. Indeed, a reduction in prescribing of immunosuppressants and biologics has been a common practice [45,46]. Generally, the decision to discontinue systemic treatment has been reported to be autonomous, due to fear of a risk of infection, rather than shared with the general practitioner. As consequence, a significant percentage of patients experience worsening of the disease, mainly because of drug withdrawal [47]. A cross-sectional study among Chinese patients with psoriasis, conducted by a web questionnaire, demonstrated that nonadherence to treatment during the COVID-19 epidemic was associated with aggravation of psoriasis, perceived stress and symptoms of anxiety and depression [48]. If patients express concerns about the safety of systemic psoriasis therapy during the COVID-19 pandemic, shared decision-making is needed after informing the patient about current guideline recommendations and about the benefits and risks of treatment discontinuation [49,50].

We acknowledge the limitations of our study which is not a systematic, but rather a narrative review of the literature. The manuscripts included in the review are heterogeneous in terms of population, treatments and measure of outcomes. The earlier studies may have been characterized by poor methodological quality. Nonetheless, our findings appear unequivocal, and although additional studies may be helpful to confirm these results, existing studies appear sufficient to conclude that psoriatic patients receiving systemic treatment are unlikely to be at increased risk of SARS-CoV-2 infection or severe COVID-19. Moreover, patients affected by inflammatory diseases, such as rheumatoid arthritis and inflammatory bowel disease, and treated with biologics show similar COVID-19 clinical outcomes to those of psoriatic patients [51]. However, these results should be confirmed by further studies comprising a longer follow-up period and including a control group. Moreover, other studies are needed to investigate the prevalence of Sars-CoV-2 infection in asymptomatic patients with psoriasis.

## 5. Conclusions

It is reasonable to interrupt systemic therapy in psoriasis patients that test positive for SARS-CoV-2 or with COVID-19 because potentially negative effects of the therapy cannot be fully excluded. Currently, however, there is no evidence to support the idea that patients receiving systemic therapy should stop their treatment. Rather, monitoring frequency could be increased in the beginning of newly started systemic therapy. To better understand the impact of SARS-CoV-2 infection, psoriasis patients that test positive should be followed carefully and entered into global registries, such as PsoPROTECT. People with psoriasis undergoing systemic therapy should be advised to follow current guidelines for hygiene and physical distancing as recommended in their respective area of residence. Given that this is a novel and rapidly changing situation, recommendations may be modified as more data become available [52]. 

## Figures and Tables

**Table 1:** Studies investigating the risk of Sars-CoV-2 infection in patients with psoriasis receiving systemic treatments.

Reference	Nationality	Type of Study	Patients, n	Treatment	Measure of Association	Results
Baniandrès-Rodrìguez et al., 2020	Spain	Multicenter prospective cohort registry based	2329	S and B DMARDs	SIR for infection	1.58 (95% CI 0.98–2.41)
Piaserico et al., 2020	Italy	Multicenter cohort	1830	Biologics	IR for infection	9.7 (95% CI 3.9–20.1)
Strippoli et al., 2020	Italy	Monocentric cohort	139	Biologics	Prevalence of infection	3.6% vs. 0.7%
Damiani et al., 2020	Italy	Case control study	1193	Biologics and apremilast	Odds Ratio	3.43 (95% CI 2.25–5.73)
Di Lernia et al., 2020	Italy	Case series	130	Cyclosporine	Number of cases	2

SIR standardized incidence rate; IR incidence rate; S and B DMARDs synthetic or biological disease-modifying antirheumatic drugs.

**Table 2:** Studies investigating the risk of hospitalization, intensive care unit admission or death due to COVID-19 in patients with psoriasis receiving systemic treatments.

Reference	Nationality	Type of Study	Patients, n	Disease	Treatment	Measure of Association	Results
Gisondi et al., 2020	Italy	Retrospective multicenter observational study	5206	PsO	Biologics	IR for hospitalization	5.6 (95% CI 1.5–14.3)
						IR for death	0 (95% CI 0–5.1)
Gisondi et al., 2020	Italy	Retrospective monocentric observational study	980	PsO	Biologics	Frequency of hospitalization	0
						Frequency of death	0
Baniandrès-Rodrìguez et al., 2020	Spain	Multicenter prospective cohort registry based	2329	PsO	s-DMARD	SIR for hospitalization,	1.55 (95% CI 0.67–3.06)
						SIR for ICU,	1.78 (95% CI 0.05–9.93)
						SIR for death	1.38 (95% CI 0.03–7.66)
Piaserico et al., 2020	Italy	Multicenter cohort	1830	PsO	Biologics	IR for hospitalization	6.5 (95% CI 11.4–11.7)
						IR for death	0 (95% CI 0–10.4)
Helcomb et al., 2020	US	Retrospective cross-sectional	412	PsO, HS, AD,	s-DMARD	Frequency of infection	5
						Frequency of hospitalization	1 (0.2) versus 10%
						Frequency of death	0
Di Lernia et al., 2020	Italy	Case series	130	PsO, AD	Cyclosporine	Frequency of hospitalization	0
						Frequency of death	0
Damiani et al., 2020	Italy	Case control study	1193	PsO	Biologics and small molecules	Risk of hospitalization (OR)	3.59 95% CI 1.49–8.63
						Risk of ICU (OR)	3.41 95% CI 0.21–54.55
						Risk of death (OR)	0.41 95% CI 0.03–6.59
Burlando et al., 2020	Italy	Retrospective monocentric observational study	515	PsO	Biologics	Frequency of hospitalization	0
						Frequency of death	0
Fougerousse A et al., 2020	France	Multicentre cross-sectional study	1418	PsO	s-DMARD	Frequency of infection	12 (0.85%)
						Frequency of hospitalization Frequency of ICU	0
						Frequency of death	5 (0.35%)
							0
Lima XT et al., 2020	US/Brazil	Retrospective registry-based study	104	PsO	s-DMARD vs no treatment	Frequency of hospitalization Frequency of ICU	15 (40.5%) vs. 26 (38.8%)
						Frequency of death	3 (5.6%) vs. 24 (35.8%)
							2 (5.6%) vs. 7 (10.8%)
Mahil et al., 2020	International	Retrospective registry-based study	374	PsO	s-DMARD	Frequency of hospitalization Frequency of death	77 (21%)
						Risk of hospitalization (biologics vs conventional)	9 (2%)
							OR 2.84, 95% CI 1.31–6.18
Yousaf A et al., 2020	US	Cohort study	53,511,836	PsO, RA, PSA, IBD, AS	MTX and anti TNF-a vs. no treatment	Likelihood of hospitalization	RR 0.91, 95% CI 0.68–1.22
						Likelihood of mortality	RR 0.87, 95% CI 0.42–1.78
Haberman R et al., 2020	US	Prospective case series	86	PsO, RA, PSA, IBD, AS	s-DMARD	Incidence of hospitalization in patients receiving biologics	6 out 72 (11%)

SIR standardized incidence rate; IR incidence rate; ICU intensive care unit; s-DMARD synthetic disease-modifying antirheumatic drugs; PsO, RA, PSA, IBD, AS: psoriasis, rheumatoid arthritis, psoriatic arthritis, inflammatory bowel disease, ankylosing spondylitis; HS hidradenitis suppurativa; AD atopic dermatitis.

**Table 3:** Studies describing the course of COVID-19 in psoriasis patients on biologics.

Reference	Nationality	Type of Study	Patients, n	Treatment	Withdrawal	Outcome
Di Lernia et al., 2020	Italy	Case report	1	Secukinumab	Yes	Fully recovered
Facheris et al., 2020	Italy	Case report	1	Ixekizumab	yes	Fully recovered
Balestri et al., 2020	Italy	Case report	1	Ixekizumab	no	Fully recovered
Gisondi et al., 2020	Italy	Retrospective multicenter observational study	3	Guselkumab Adalimumab	yes	Fully recovered
				Ustekinumab		

Kiss et al., 2020	Hungary	Case report	1	Risankizumab	yes	Fully recovered
Carugno et al., 2020	Italy	Case report	1	Secukinumab	yes	Fully recovered
Magnano et al., 2020	Italy	Case series	9	Ixekizumab (2) Guselkumab	yes	Fully recovered
				Secukinumab Adalimumab (3)		
				Ustekinumab		
				Etanercept		
Messina et al., 2020	Italy	Case report	1	Guselkumab	yes	Fully recovered
Benhadou et al., 2020	Belgium	Case report	1	Guselkumab	no	Fully recovered
Silva et al., 2020	Spain	Case series	7	Apremilast (3)	No (apremilast)/yes	Fully recovered
				Secukinumab (2)		
				Infliximab (2)		
Vispi et al., 2020	Italy	Retrospective monocentric observational study	246; 1	Ustekinumab	No	Fully recovered
Conti et al., 2020	Italy	Case series	4	Adalimumab	No (adalimumab)/yes	Fully recovered
				Ustekinumab		
				Secukinumab Guselkumab		
Brownstone et al., 2020	US	Case series	2	Adalimumab	yes	Fully recovered
				Ustekinumab		
Strippoli et al., 2020	Italy	Monocentric cohort study	139; 5	Infliximab	yes	Fully recovered
				Etancercept Adalimumab Ustekinumab Ixekizumab		
Mugheddu et al., 2020	Italy	Case report	1	apremilast	No	Fully recovered
